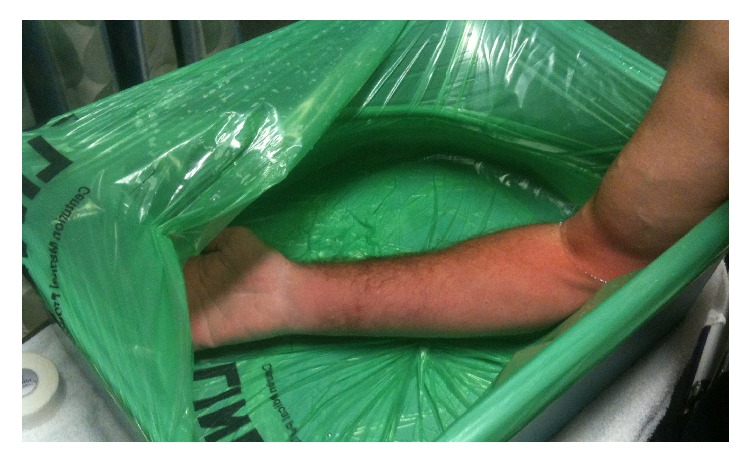# A Case of Lionfish Envenomation Presenting to an Inland Emergency Department

**DOI:** 10.1155/2017/5893563

**Published:** 2017-08-13

**Authors:** Rachel F. Schult, Nicole M. Acquisto, Crystal K. Stair, Timothy J. Wiegand

**Affiliations:** ^1^Department of Pharmacy, University of Rochester Medical Center, 601 Elmwood Ave. Box 638, Rochester, NY 14642, USA; ^2^Department of Emergency Medicine, University of Rochester Medical Center, 601 Elmwood Ave. Box 655, Rochester, NY 14642, USA; ^3^Critical Care Medicine, Department of Pharmacy, Yale New Haven Hospital, 20 York Street, New Haven, CT 06510, USA

## Abstract

Lionfish envenomation can cause erythema, edema, necrosis, and severe pain at the exposed site. Treatment often includes supportive wound care, pain management, and hot water immersion. We report a case of lionfish exposure presenting to an inland emergency department treated successfully with these measures.

## 1. Introduction

Lionfish (*Pterois volitans*, family Scorpaenidae) and other venomous fish are frequently imported for home aquariums making envenomation possible in areas outside of indigenous coastal locations. There are estimated 40,000–50,000 marine fish envenomations that occur annually throughout the world with stingrays being the only exposure to occur more frequently than Scorpaenidae envenomation [1]. Although the majority of cases occur after encounter in their native habitat, most notably the tropical Indo-Pacific region, multiple reports exist of exposures in nonindigenous areas making it important for all emergency medicine clinicians to understand treatment [2, 3]. Venom is released from bony spines along the fins after puncturing the skin of the victim, which frequently occurs during attempted handling or cleaning of the aquarium. Typical symptoms include immediate pain followed by erythema and edema. Severe envenomation is associated with local necrosis developing over several days [2]. We report a case of lionfish envenomation presenting to an inland emergency department (ED) following home aquarium exposure.

## 2. Case Presentation

A 49-year-old male with no prior medical history presented to the ED approximately 4 hours after being stung on the right forearm by a lionfish while cleaning his home salt-water aquarium. The patient reported that he felt a sting followed by immediate pain and bleeding from the puncture site. He became diaphoretic with continued pain and experienced minimal relief after removing the barbs from the puncture site, taking aspirin and ibuprofen, and soaking his arm for 2 hours in a warm water bath. The patient presented to the ED for further pain management.

**Figure 1:**
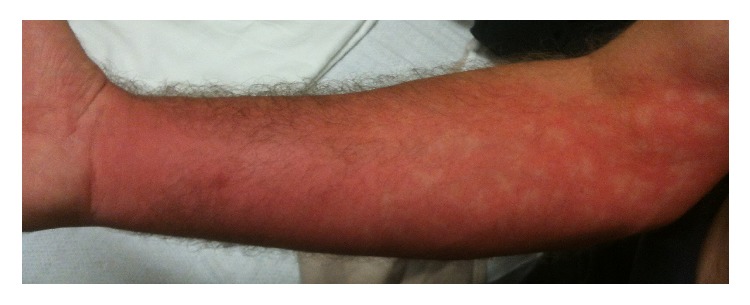


On initial exam the patient was noted to have erythema, edema, and urticaria extending up the right arm from his fingertips to the lower portion of the humerus, with a visible puncture wound at the volar aspect of the forearm (Figure 1). He reported continued burning pain, described as 10/10 pain, although he did feel that it had plateaued since exposure. He denied chest pain, palpitations, shortness of breath, lightheadedness, or nausea/vomiting/diarrhea. His initial blood pressure was 139/95 mmHg, heart rate 105 beats/minute, respiratory rate 24 respirations/minute, and temporal temperature 37.2°C.

The medical toxicology service was consulted and the patient was treated per their recommendations. The extremity was submerged in a hot water bath for 30 minutes with subjective, immediate improvement in symptoms (Figure 2). After 10 minutes of the water bath, the patient also received parenteral hydromorphone 1 mg every hour for 4 hours for continued pain. An X-ray performed to evaluate for residual foreign body was negative, and tetanus vaccination was administered. The patient had improvement in pain and was admitted to the observation unit for overnight monitoring and continued pain management with parenteral analgesics.

The following morning, 6 hours after the last dose of analgesic medication, the patient's pain score was reported as 0/10 and the swelling and erythema were improved. He received a total of 4 mg hydromorphone and 10 mg oxycodone during his hospitalization. All laboratory values, including creatine kinase, remained within normal limits and the patient was discharged home approximately 20 hours after envenomation.

## 3. Case Discussion

Lionfish belong to the Scorpaenidae venomous family of fish, also containing scorpionfish and stonefish. Lionfish venom is the least potent in this family [1]. Local pain at the site of envenomation, most commonly described as throbbing, may progress to involve the entire extremity. Pain typically peaks within 60–90 minutes and continues up to 12 hours, although it may be present for days or even weeks [1, 2]. Wound severity is classified into three categories with grade I being least severe and consisting of local erythema or ecchymosis surrounding the wound. Grade II envenomations consist of vesicle or blister formation, and grade III includes wounds developing local necrosis over the ensuing days to weeks [1, 4].

Treatment of lionfish envenomation involves supportive care with local wound management, tetanus prophylaxis, and pain control. Antibiotics are not recommended unless infection is present. Because the predominant toxin found in lionfish venom is heat labile, the mainstay of therapy is centered on hot water immersion of the affected area. Ideally, water should be as hot as tolerated but nonscalding (up to 45°C) and the extremity should remain in the bath for 30–90 minutes. In the event that pain is not controlled with hot water bath and analgesics, nerve block with 0.25% bupivacaine has been reportedly effective and may be considered if feasible [5]. Nerve block should likely not be combined with hot water immersion due to the risk of thermal burn if the water temperature is too high.

Several articles have detailed poison control center (PCC) experience with venomous fish stings after exposure from domestic aquariums [2–4]. The largest series documented 45 lionfish envenomations over a 5-year period with 82% occurring at home and the remainder occurring in tropical fish stores [2]. Importantly, 73% of cases were reported to the PCC after patient presentation to an ED. All patients reported immediate pain at the site of the sting and 22% of patients experienced pain in most or all of the affected extremity. No patients developed grade III envenomation; however, wound infections occurred in four patients. Systemic toxicity is rarely reported but may include nausea, diaphoresis, shortness of breath, chest pain, weakness, or hypotension; of these, nausea was most commonly reported and occurred in 13% of patients. Of 35 patients with follow-up information available, 80% reported complete resolution of pain shortly after hot water immersion; there were no patients with persistent pain after 24 hours. Other reports from inland PCCs have documented similar results; exposures occur predominantly in the home, local pain is the primary complaint, and few cases of secondary cellulitis are encountered [3, 4]. Although our patient presented four hours after exposure with symptoms consistent with grade I envenomation, he had signs of systemic toxicity (diaphoresis) and persistent pain not improved with home remedies. Hot water immersion was effective and he was discharged the following morning without persistent symptoms.

## 4. Conclusion

We report a case of lionfish envenomation presenting with severe pain to an inland ED after exotic aquarium sanitation in the home treated successfully with hot water immersion and parenteral pain control. Although frequently encountered near indigenous tropical locations, emergency medicine clinicians in all locations should be familiar with management due to possible exposures from exotic aquariums.

## Figures and Tables

**Figure 2:**